# Nocturnal Incontinence of Children

**Published:** 1890-08-02

**Authors:** 


### THE PRACTITIONER'S MIRROR AND RETROSPECT.

[*The Editor will be glad to receive offers of co-operation and contributions from members of the profession. There is no doubt that much valuable material full of interest and instruction is at present lost to science. This is largely so in the case of* (1) *the younger and less-known members of the hospital staff* (2) *those connected with cottage hospitals, and* (3) *the great body of medical practitioners not attached to any institution. The co-operation of all is cordially invited to enable the Editor to make* The Hospital *of the utmost possible use and value to the profession. Specialists willing to review books on their special subjects are invited to communicate this fact at once. All letters should be addressed to* The Editor, The Lodge, Porchester Square, London, W.]

### MEDICINE.

### NOCTURNAL INCONTINENCE OF CHILDREN.

Dr. W. A. Briggs, of Sacramento, contributes to the *Occidental Medical Times* a note on nocturnal incontinence in children. Many theories have been broached to account for this affection. Some have believed it to be chiefly nervous, many children so affected inheriting neurotic tendencies. This appears to be the author's opinion, as he thinks electricity applied by Guyon's method offers the most hope of relief. Its use requires an insulated sound with a bulbous metallic extremity. One pole is connected with this, and the other applied either to the pubes or the perineum. The sound is introduced to the neck of the bladder, the current turned on very gently, while the sound is drawn back a little into the urethra and then returned to the bladder. The first sitting should not exceed five minutes, and later ones ten, and the bladder should contain some water. Now we are by no means satisfied that wetting the bed by children is most frequently chiefly nervous. Indeed, we are of opinion that it most generally arises from other causes. It may depend on phimosis, on uric acid, on worms, especially thread worms, or a stone in the bladder. The treatment, therefore, consists in finding out and attacking the cause. If there is no evidence of maladies as above, and there is also "night screaming," bromides may be given with advantage. In cases where there is no assignable cause, the child should be made to empty the bladder immediately before going to bed, and also be provided with a vessel which may be taken into bed. If the practice is persisted in, either idly or unconsciously, he should be roused in the middle of the night for the purpose of emptying the bladder. He should also be induced to hold the water as long as possible in the day time, so that the bladder may become accustomed to being full; and in the night to lie on the side, and not on the back, and to prevent his turning, an ordinary cotton reel may be fixed behind. As a general rule, as the child grows older the habit is discontinued, and we think manipulation with instruments, as recommended by Dr. Briggs, can rarely be required, and may often be very injurious.

